# Patterns of ferns community assemblages in some Malaysian and Nigerian tropical forests

**DOI:** 10.1002/ece3.8961

**Published:** 2022-06-03

**Authors:** Gbenga F. Akomolafe, Rusly Rosazlina, Zakaria Rahmad, Fatai A. Oloyede

**Affiliations:** ^1^ 26689 School of Biological Sciences Universiti Sains Malaysia, 11800 Minden Penang Malaysia; ^2^ Department of Plant Science and Biotechnology Federal University of Lafia Lafia Nigeria; ^3^ 54715 Department of Botany Obafemi Awolowo University Ile‐Ife Nigeria

**Keywords:** ecological zones, ferns diversity, intercontinental, species richness, tropical forests

## Abstract

Research on fern ecology has gained attention in the last decade, yet there is a paucity of information on the comparison of ferns communities across continents. This study focused on comparing the ferns community assemblages in some tropical forests of Malaysia and Nigeria, thereby assessing the patterns of species richness (SR) and phylogenetic diversity (PD) in relation to the bioclimatic drivers across the continents. The diversity and taxonomic compositions of ferns were assessed using 180 plots of 10 m × 10 m in each country. The species richness and other diversity indices were determined using the combined forests data for each country and for the individual forests. Also, the phylogenetic diversity of the ferns was assessed using the genus‐based molecular sequences downloaded from the GeneBank. The patterns of the ferns SR and PD in the two countries as driven by some bioclimatic factors were evaluated using the regression analysis. The observed and rarefied–extrapolated fern species richness is significantly higher in Malaysian forests than in Nigerian forests. Also, the other diversity indices are significantly higher in Malaysian forests except for the Shannon index which showed no significant difference between the two biogeographic regions. There is a very low similarity (7.41%) in the taxonomic composition of ferns between the two biogeographic areas, although the similarity in composition increased with increasing taxonomic levels (species: 7.41%, genus: 12.77%, family: 70.96%). Terrestrial and epiphytic ferns are more dominant than the other life forms in the two countries. The precipitation variables drive the phylogenetic structure of ferns in Nigeria, whereas both precipitation and temperature variables drive the phylogenetic structure of ferns in Malaysia. This indicates that ferns assemblages in Nigeria and Malaysia are driven by both climatic variables. Besides, we also hypothesize that these observed differences could be due to other historical and evolutionary processes.

## Graphical abstract

The study assessed the patterns of ferns species richness (SR) and phylogenetic diversity (PD) in relation to the bioclimatic drivers in some Nigerian and Malaysian forests. Fern species richness and diversity is significantly higher in Malaysian forests than in Nigerian forests. The precipitation variables drive the phylogenetic structure of ferns in Nigeria, whereas both precipitation and temperature variables drive the phylogenetic structure of ferns in Malaysia.

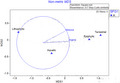

## 1 INTRODUCTION

Ferns are known to be an essential part of the biodiversity and vegetations of tropical forest ecosystems (Haque et al., 2016). They originated from the old world tropics and have colonized other regions of the world (Mehltreter et al., 2010). Ferns generally constitute the substantial biomass of many tropical and subtropical forests of the world (Yusuf, 2010). Their occurrence and abundance in these tropical regions are largely dependent on moisture availability (Dixit, 2000). Apart from their biodiversity roles in the ecosystems, they are also useful to mankind in diverse ways such as for ornamentals, medicines, and food (Sarker & Hossain, 2009; Uddin et al., 2008). Despite the ecological and economic importance of ferns, studies on comparing ferns communities across regional and continental scales are largely scarce (Salazar et al., 2015). Most ecological studies on the patterns of species diversity and assemblages across larger spatial scales have been focused on higher plants and vertebrates (Fritz & Rahbek, 2012; Weigelt et al., 2015).

Over the years, there have been several collections of ferns across many tropical forests of Malaysia which have led to the documentation of over 1165 fern species of the 4400 species reported for South East Asia (Rahmad & Akomolafe, 2019). Similarly, ferns are known to be found widely across many ecological zones of Nigeria, constituting about 165 species, 64 genera, and 27 families (Akinsoji et al., 2016). However, several factors such as unplanned urbanization, farming, and mineral exploitations have threatened their existence in many parts of Nigeria (Akomolafe & Sulaimon, 2018). Malaysia and Nigeria are both located within the tropics, yet they both differ in some climatic conditions. For instance, researchers have reported that tropical forests in Africa receive lower amount of rainfall which is usually between a specific period between May and September, than the ones of Southeast Asia which is almost throughout the year (Addo‐Fordjour & Rahmad, 2015; Malhi & Wright, 2004). This results in a higher relative humidity in Asian tropical forests than in African tropical forests (Lewis et al., 2013). These climatic differences have caused variations in the floristic composition between the two regions. They also differ in geographic and topographic aspects (e.g., island vs. mainland region, differences in elevational ranges), which are important for fern diversity patterns (Lwanga et al., 1998). However, there is little information on the ferns differences between Asian and African forests as researchers are more focused on trees, lianas, and other plant types (Addo‐Fordjour et al., 2016).

Due to the evolutionary and climatic differences of the two continents, single research that assesses floristic data from these two tropical regions can boost our understanding of biogeographic patterns in fern ecology. Research that covers more than one continent is regarded as more effective in drawing the intercontinental patterns of species ecology than individual researches that only focus on one specific continent (Addo‐Fordjour et al., 2016). Hence, our study focused on determining the patterns of ferns community assemblages in some Malaysian and Nigerian tropical forests.

Besides assessing the diversity patterns of the ferns across the two countries, it is also important to understand the differences in the diversity patterns of the forests within each country. This is crucial because the individual forests in each country are in different habitat types and under different conservation and preservation statuses. For instance, within each country, this study focused on university campus forest which has been drastically encroached by humans and reserved forest which is strictly under protection. The levels of anthropogenic disturbance in the university campuses are expected to impact negatively on the plant diversity due to the removal of vegetation cover of the forests to enable infrastructural developments. On the contrary, it is expected that the reserved forests would accommodate a higher diversity of plants due to the restriction in anthropogenic disturbances.

Incorporating phylogenetic data into biodiversity researches has been described as a promising way of providing long‐term evolutionary perspectives (Cavender‐Bares et al., 2009; Emerson & Gillespie, 2008). Therefore, it is necessary to compare the ferns community assemblages between Malaysian and Nigerian tropical forests from both taxonomic and phylogenetic aspects as these offer different insights into community assembly. Phylogenetic analysis has been increasingly used by community ecologists to generate temporal dimensions which help in drawing reasonable conclusions to diversity patterns of plants assemblages (Cadotte et al., 2010).

Consequently, the questions asked were as follows: (1) Do fern diversity and community structure differ between Malaysian and Nigerian forests? (2) Are there any similarities in the ferns taxonomic composition between Malaysian and Nigerian forests? (3) How do fern diversity and community structure differ within each country? (4) Is there any difference in the phylogenetic structure of the ferns community between the two Countries? (5) Are the observed ferns phylogeny and species richness driven by any bioclimatic factors?

## 2 MATERIALS AND METHODS

### 2.1 Study area

**FIGURE 1:**
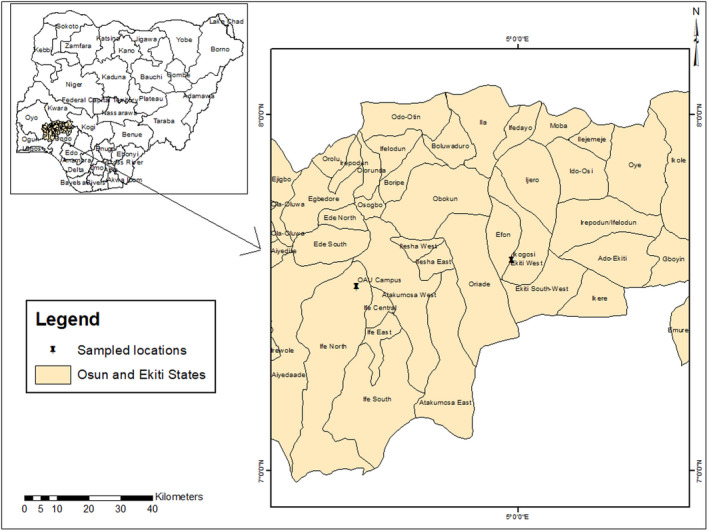
Study area map of Nigeria showing OAU campus and Ikogosi warm spring forest

The study was conducted in some tropical recreational forests and University campuses in Nigeria and Malaysia. In Nigeria, the fern species were collected from the campus of the Obafemi Awolowo University (OAU), Ile‐Ife, Osun State and the reserved forest near Ikogosi warm spring, Ekiti State (Figure 1). This University campus is located in Ile‐Ife which lies between latitude 7°32′N, longitude 4°32′E and elevation 258 m. The campus was originally sited on a primary forest which has now been transformed into secondary forest due to the level of human encroachments (Oloyede et al., 2014). Ile‐Ife city falls within the tropical rain forest zone of Nigeria which receives an average annual rainfall of 1400 to 1500 mm and a mean annual temperature ranging between 27°C and 34°C. The tropical rain forest vegetation is characterized mainly by abundant trees with few woody shrubs. Ile‐Ife like other cities in Nigeria has two main seasons including the dry and rainy seasons. The city experiences rainy season between March and October, whereas it experiences dry season between November and early March (Oke & Isichei, 1997). The second site, Ikogosi warm spring forest is located at Ikogosi, Ekiti State. It has a geographical boundary of latitude 7°34′N, longitude 4°58′E, and elevation 492 m. This warm spring has been recognized as a recreational center which houses both primary and secondary tropical rain forests.

**FIGURE 2:**
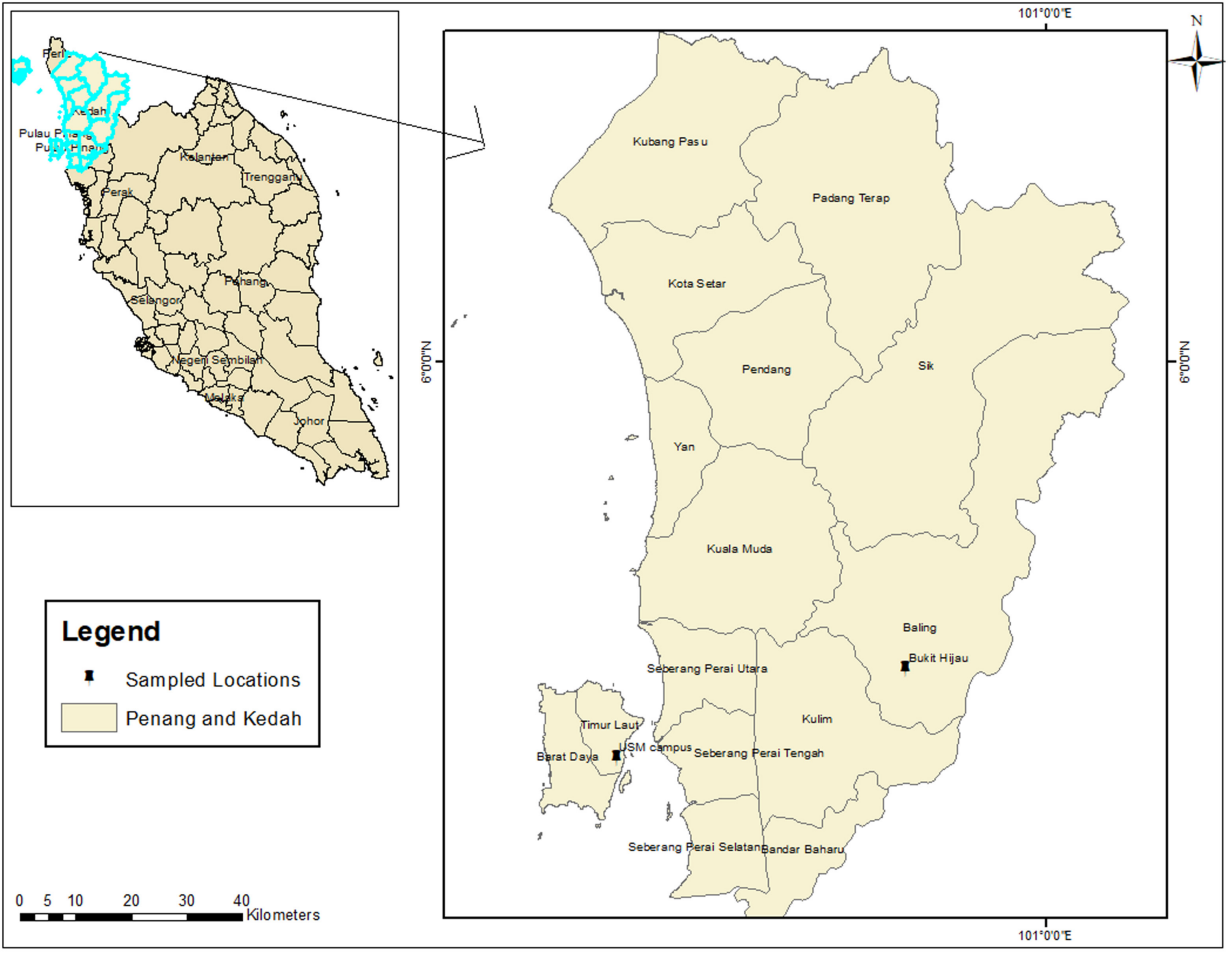
Study area map of Peninsular Malaysia showing USM campus and Bukit Hijau forests

In Malaysia, the ferns were collected from the main campus of the Universiti Sains Malaysia (USM), Penang Island and Bukit Hijau recreational forest, Kedah (Figure 2). Both sites are located within the Peninsular Malaysia. This peninsular is known to be the floristically richest part of Indomalesian sub‐kingdom (Rahmad & Akomolafe, 2018). This campus located on latitude 5°21′N, longitude 100°18′E, and elevation 10 m has about 252.7 ha of land. The vegetation comprises extensively large canopy trees of both primary and secondary forests. This city has a tropical climate with an average annual rainfall of almost 2670 mm (Rahmad et al., 2014). Also, it receives a daily temperature range of 24°C to 32°C and relative humidity of 70%–90%. The second site which is Bukit Hijau recreational forest is located at 42 km away from Baling town, Kedah, Peninsular Malaysia. It has a geographic boundary of latitude 5°30′N and longitude 100°46′E. This forest was established as a recreational forest in 1959, and it has been used for activities such as picnics, campsites, eco‐tourism, and educational activities (Rahmad & Akomolafe, 2019). The forest has also housed wildlife, such as elephants, tigers, tapirs, monkeys, deer, birds, and squirrels (Hussein, 2014). The forest is characterized by lowland and slightly hill dipterocarp vegetation with a waterfall and is elevated at 150–300 m above sea level.

### 2.2 Ferns sampling technique

In each site, ferns were sampled at three different areas within the forested areas. This includes the undisturbed area, less‐disturbed area, and most‐disturbed area. These areas were selected based on the observation of the rate of human encroachments and infrastructural developments. The sampling was designed to cover all the three areas. Thirty plots of size 10 m × 10 m were established in each area, giving rise to a total of 90 plots per site and 180 plots in each country. A preferential nonrandom method of sampling, which ensured that at least one individual fern was captured in each plot, was adopted for the study (Akinsoji et al., 2016). In this sampling, the plot was established preferentially by placing it where a fern was found. This means that no plot had an absence of a fern species. This was to ensure that the fern species in the sites were properly captured.

In all the established plots, the abundance of individual ferns found was noted and categorized as terrestrial, aquatic, epiphytes, and lithophytes. Some of the ferns were identified directly on the field. Those with difficult identification were pressed and identified at the herbaria of Universiti Sains Malaysia and Federal University of Lafia. The epiphytes were sighted using the field binoculars while some big trees were climbed to collect epiphytes with difficult on‐site identification. The fern species were thereafter identified using taxonomic flora (Parris et al., 2010; Parris et al., 2013; Parris et al., 2020; Piggott, 1988), and their names were verified using an online database of International Plant Names Index. The classification system of Pteridophyte Phylogeny Group (PPG) (2016) was used for the species list. The voucher specimens were deposited in the respective herbarium depending on the country for references. The conservation status of each identified fern was assessed from the redlist database of the International Union for the Conservation of Nature (IUCN) (www.iucnredlist.org).

### 2.3 Climatic drivers of the ferns distribution

The key climatic factors that have been identified as drivers of plants species distribution across large spatial scales include the precipitation seasonality, precipitation during the driest month, minimum temperature of the coldest month, annual precipitation, mean annual temperature, and temperature seasonality (Kooyman et al., 2012). The distribution of plant species is further influenced by extreme climatic conditions such as the seasonal variations in temperature and precipitation (Weigelt et al., 2015). The climatic variables were gotten from the WorldClim website at a resolution of 30 arc‐s. The bioclimatic variables used are coded as bio1, bio12, bio4, bio14, bio6, and bio15. The temperature variables include the bio1, bio6, and bio4 while the precipitation variables include the bio12, bio14, and bio15 (Qian et al., 2021).

### 2.4 Molecular data and phylogenetic analyses

The Genus‐based phylogenetic tree was used to understand the evolutionary relationship of the ferns in each continent. This is because we could not get access to the molecular sequences of all the fern species for building the species‐based phylogenetic tree. The sequences used for each fern genus were gotten from the Genbank (https://www.ncbi.nlm.nih.gov/). Three markers were initially selected (atpA, atpB, and rbcL), but we finally used rbcL due to it having the lowest amount of missing data. The sequences were aligned and they were used to construct a maximum likelihood phylogeny using the MEGA (Molecular Evolutionary Genetics Analysis) version 11. Faith's phylogenetic diversity (PD: Faith, 1992) which measures the phylogenetic diversity of the fern species assemblage in each country of study was determined by summing all the phylogenetic branch lengths that connects the genus in the phylogenetic tree of each country. This PD has been stated as strongly correlated with the species richness of an assemblage (Forest et al., 2007).

### 2.5 Data analyses

The relative frequency of each fern species was calculated for the two biogeographic areas. To have a considerable comparison of the species richness between the two campuses, a nonasymptotic rarefaction‐extrapolation analysis which is a species richness evaluator was employed (Addo‐Fordjour et al., 2016). This was performed using the individual‐based abundance data for each fern species. Significant difference in the rarefied–extrapolated fern species richness between the countries was determined by the confidence intervals of the species accumulation curves, constructed using 50 bootstrap replicates. This analysis was achieved with the aid of iNEXT software (online version) (Chao et al., 2016). An overlap in the confidence intervals of the curves indicates the absence of statistical significance difference between the species richness of the two countries. Significance difference in the species richness is only established when the confidence intervals of the curves are nonoverlapping (Rahmad & Akomolafe, 2019). This analysis is very important as it eradicates bias which is usually encountered in field sampling. The ferns diversity indices such as Shannon index, Simpson index, Evenness index, Fisher's alpha, and Margalef index were quantified for each country using paleontological statistics (PAST) 3.0 software. Fisher's alpha describes mathematically the relationship between the number of species and the number of individuals in those species (Fisher et al., 1943). Margalef's index is a species richness index which compensates for the effects of sample size through dividing the number of species in a sample by the natural log of the number of organisms collected (Death, 2008). Pairwise permutation tests were used to determine the significant differences in the diversity indices between the countries.

Sorensen's similarity index was used to determine the intercontinental similarity in the ferns compositions at the three taxonomic levels (species, genus, and family) between the two studied areas. The life forms of the ferns were associated with the two countries using a nonmetric multidimensional scaling (nMDS) analysis with Bray‐Curtis dissimilarity measures with the aid of the PRIMER 7 software. Variations in the distribution of the ferns species across the two continents were determined using the principal component analysis (PCA) of the PAST software.

Pearson's correlation analysis was used to evaluate the relationship between the PD, species richness (SR), and the bioclimatic variables for each country. The p‐values were not reported; instead, we used the strength of the correlation coefficients (Hawkins et al., 2011). The correlation coefficient (r) that is greater than 0.6 was considered strong while the one that is less than 0.3 was considered weak (Qian et al., 2019). The analysis was made using the Paleontological Statistics (PAST) software version 3.0.

## 3 RESULTS AND DISCUSSION

### 3.1 Cross‐continental comparison of ferns diversity patterns

**FIGURE 3:**
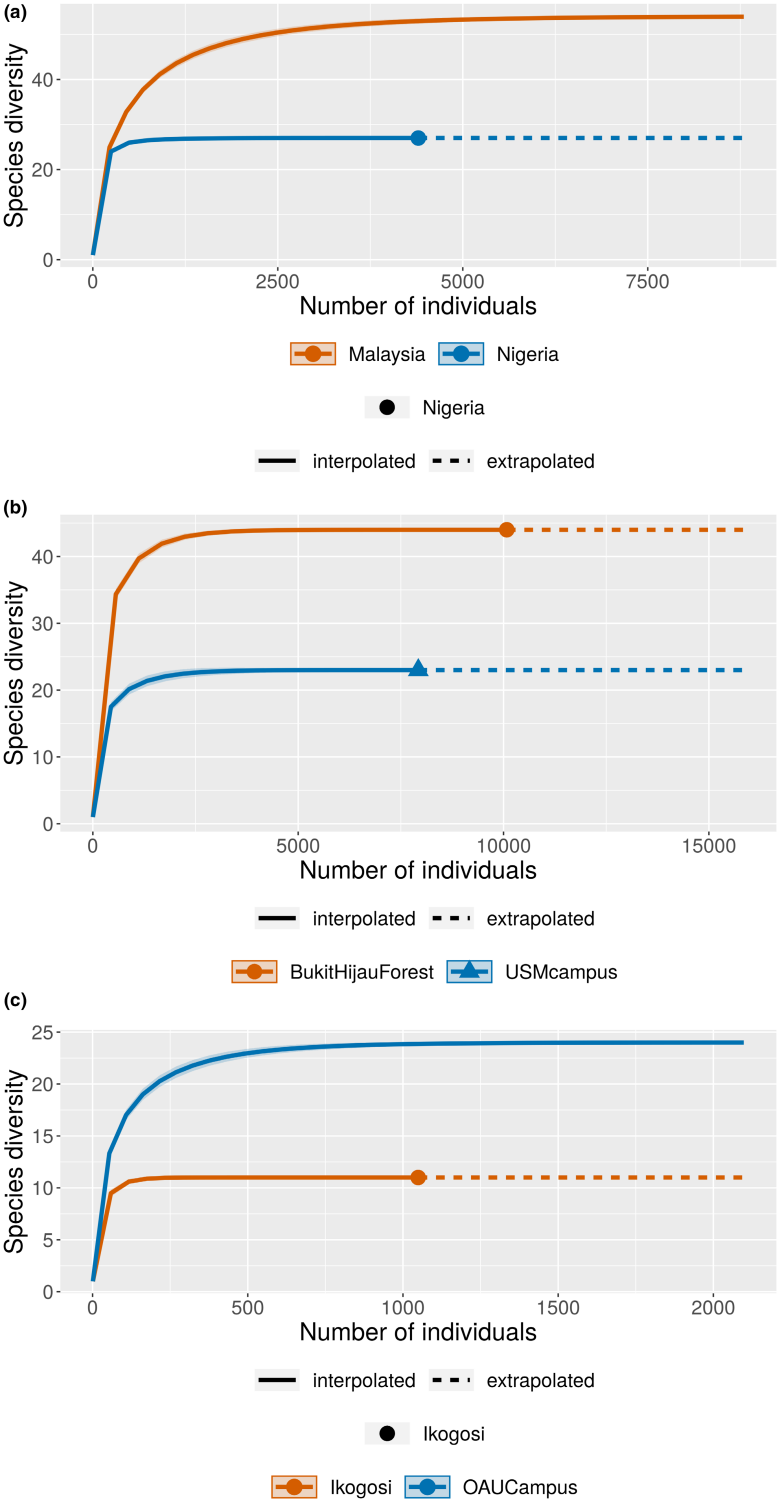
(a) Individual‐based rarefied–extrapolated species richness curves for the combined forests of both Malaysia and Nigeria. (b) Individual‐based rarefied–extrapolated species richness curves for the individual forests in Malaysia. (c) Individual‐based rarefied–extrapolated species richness curves for the individual forests in Nigeria

A total of 54 ferns species were observed in Malaysian forests while total of 27 ferns were observed in Nigerian forests. This difference in the ferns species is significant as revealed by the pairwise permutation test (*p* = .001). In the same vein, the rarefied–extrapolated species richness shows that the Malaysian forests are richer in ferns than Nigerian forests (Figure 3a). The rarefied and extrapolated species richness curves for both countries reached asymptote showing that there was an adequate sampling of the ferns in the forests. This is also evident by the equal values of the observed and rarefied–extrapolated species richness of ferns of both countries. All the diversity indices of the combined forests of Malaysia are significantly higher than those of Nigerian forests (pairwise permutation test: *p* = .001) except the Shannon diversity index which showed no significant difference (*p ˃* .05). These trends generally indicated that Malaysian forests are richer and more diverse in fern species than the Nigerian ones. This could be due to the significant roles moisture and other microclimatic conditions play in the occurrence and distribution of ferns in tropical forests (Dixit, 2000). South East Asian countries particularly Malaysia is known to receive more rainfall throughout the year than West African ones (Addo‐Fordjour et al., 2016; Lewis et al., 2013). This may account for the differences in the richness of ferns between the two continents. It is also important to state that the two countries are highly diverse in fern species. This is because ecosystems with Shannon index of 2 and above are considered highly diverse (Barbour et al., 1999; Table 1).

**TABLE 1:** Comparison of ferns community characteristics between Malaysia and Nigeria

Community characteristics	Malaysia	Nigeria
Total observed species richness*	54^a^	27^b^
Rarefied and extrapolated species richness**	54^a^	27^b^
Number of individuals	18,004	4399
Simpson index*	0.849^a^	0.826^b^
Shannon diversity index*	2.399^a^	2.345^a^
Evenness index*	0.204^a^	0.386^b^
Margalef index*	5.409^a^	3.099^b^
Fisher's alpha*	6.859^a^	3.832^b^

Note

Values with the same superscript across same row are not significantly different and vice‐versa.

* Significant differences determined by pairwise permutation tests in PAST.

** Significant difference determined by confidence interval of the curves.

### 3.2 Within country comparison of ferns diversity patterns

As for the individual forests in Malaysia, it was observed that the Bukit Hijau forest has a significantly higher number of ferns (44) than the USM campus forest (23). This trend was also observed in all the diversity indices of the two forests except for the evenness index which showed no significant difference between them (Table 2 and Figure 3b). In Nigeria, OAU campus forest was observed to have a significantly higher number of ferns (24) than Ikogosi warm spring forest (11). The same trend was also observed in all other diversity indices measured except the Simpson index (Table 2 and Figure 3c). These observed differences between the individual forests of each country could be due to other factors than climate. This is because the two study sites in each country are influenced by almost similar climatic conditions with slight differences. The most suspicious factor could be the extent of human disturbances in each forest (Akinsoji et al., 2016). For example, the Ikogosi warm spring forest is a recreational forest which receives so many tourists daily with no strict protection of the forest species. The reverse is the case for Bukit Hijau forest which has some measures of protection for its forest species (Rahmad & Akomolafe, 2019).

**TABLE 2:** Comparison of ferns community characteristics between individual sampling sites in Malaysia and Nigeria

Community characteristics	USM campus forest	Bukit Hijau forest
*Malaysia*		
Total observed species richness*	23^a^	44^b^
Rarefied and extrapolated species richness**	23^a^	44^b^
Number of individuals	7925	10,079
Simpson index*	0.762^a^	0.874^b^
Shannon diversity index*	1.828^a^	2.517^b^
Evenness index*	0.271^a^	0.282^a^
Margalef index*	2.450^a^	4.665^b^
Fisher's alpha*	2.907^a^	5.913^b^

Community characteristics	OAU Campus forest	Ikogosi warm spring forest
*Nigeria*		
Total observed species richness*	24^a^	11^b^
Rarefied and extrapolated species richness**	24^a^	11^b^
Number of individuals	3327	1049
Simpson index*	0.753^a^	0.778^a^
Shannon diversity index*	2.100^a^	1.829^b^
Evenness index*	0.340^a^	0.566^b^
Margalef index*	2.836^a^	1.438^b^
Fisher's alpha*	3.499^a^	1.714^b^

* Significant differences determined by pairwise permutation tests in PAST.

** Significant difference determined by confidence interval of the curves.

### 3.3 Patterns of life forms of ferns between the two countries

**FIGURE 4:**
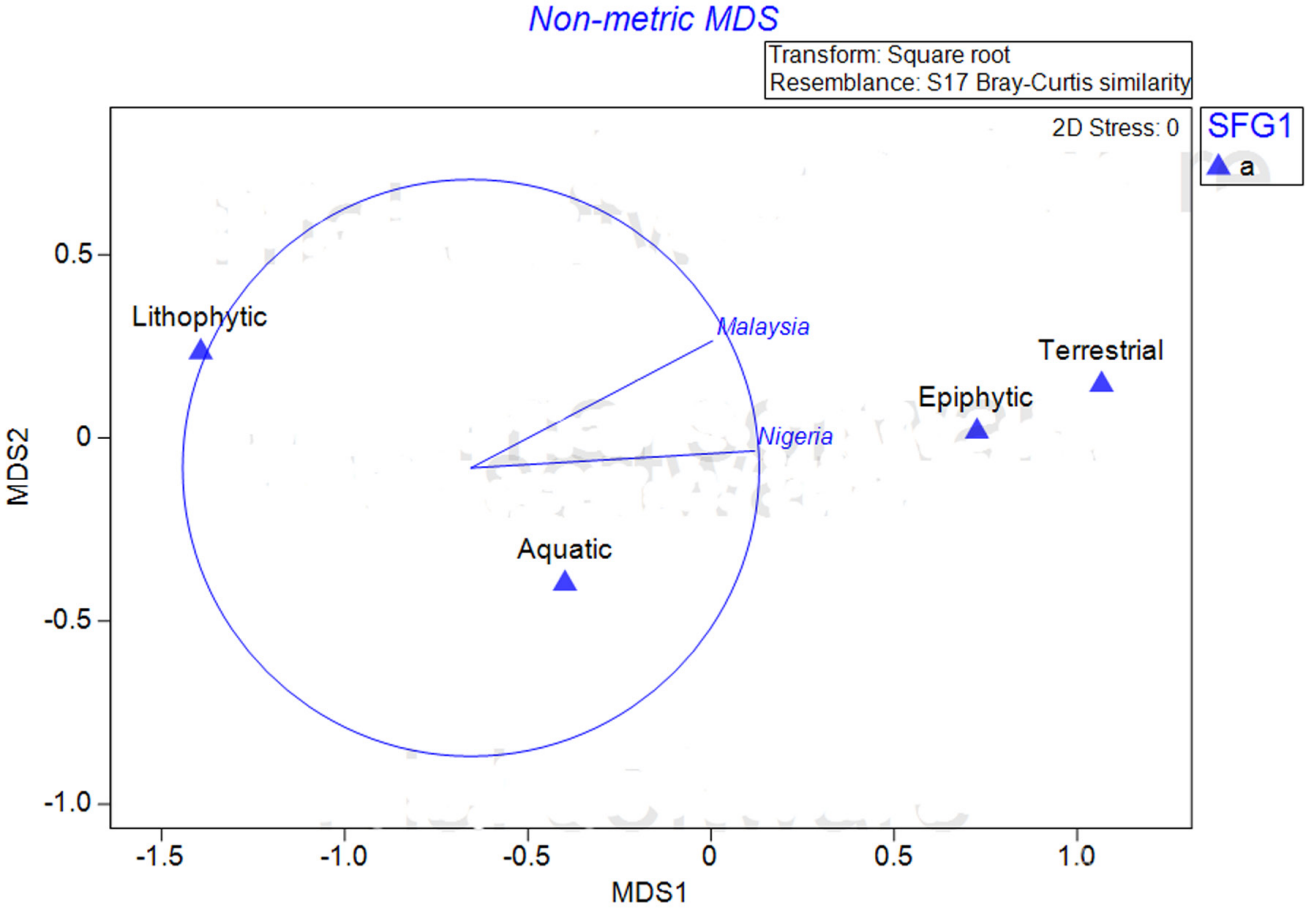
nMDS showing the association between the ferns life forms and the two Countries

Generally, terrestrial ferns are the dominant ferns in Malaysian and Nigerian forests having 37 and 17 species, respectively, while aquatic ferns are the least (Table 3). The dominant nature of the terrestrial ferns could also be an indicator to the lesser degree of disturbance of the forests in the two countries when considered on a larger scale. Researchers have confirmed the dominant nature of terrestrial ferns in less‐disturbed forests (Keerthi et al., 2001; Oloyede et al., 2014; Rahmad & Akomolafe, 2018; Sathapattayanon & Boonkerd, 2006). The nonmetric multidimensional scaling analysis showed that the Malaysian and Nigerian forests are more associated with terrestrial and epiphytic ferns than the other life forms of ferns (Figure 4). The nMDS, which is an indirect gradient analysis, usually produces ordination based on a dissimilarity matrix and separates the entities in a low‐dimensional space according to ranks. Accordingly, the terrestrial and epiphytic ferns are closer to the two Countries in this study. This explains why they are the dominant ferns in the two Countries.

**TABLE 3:** Life forms of the ferns in Malaysia and Nigeria

Life form	Number of species	
	Malaysia	Nigeria
Aquatic	2	2
Epiphytic	12	8
Lithophytic	3	0
Terrestrial	37	17

**FIGURE 5:**
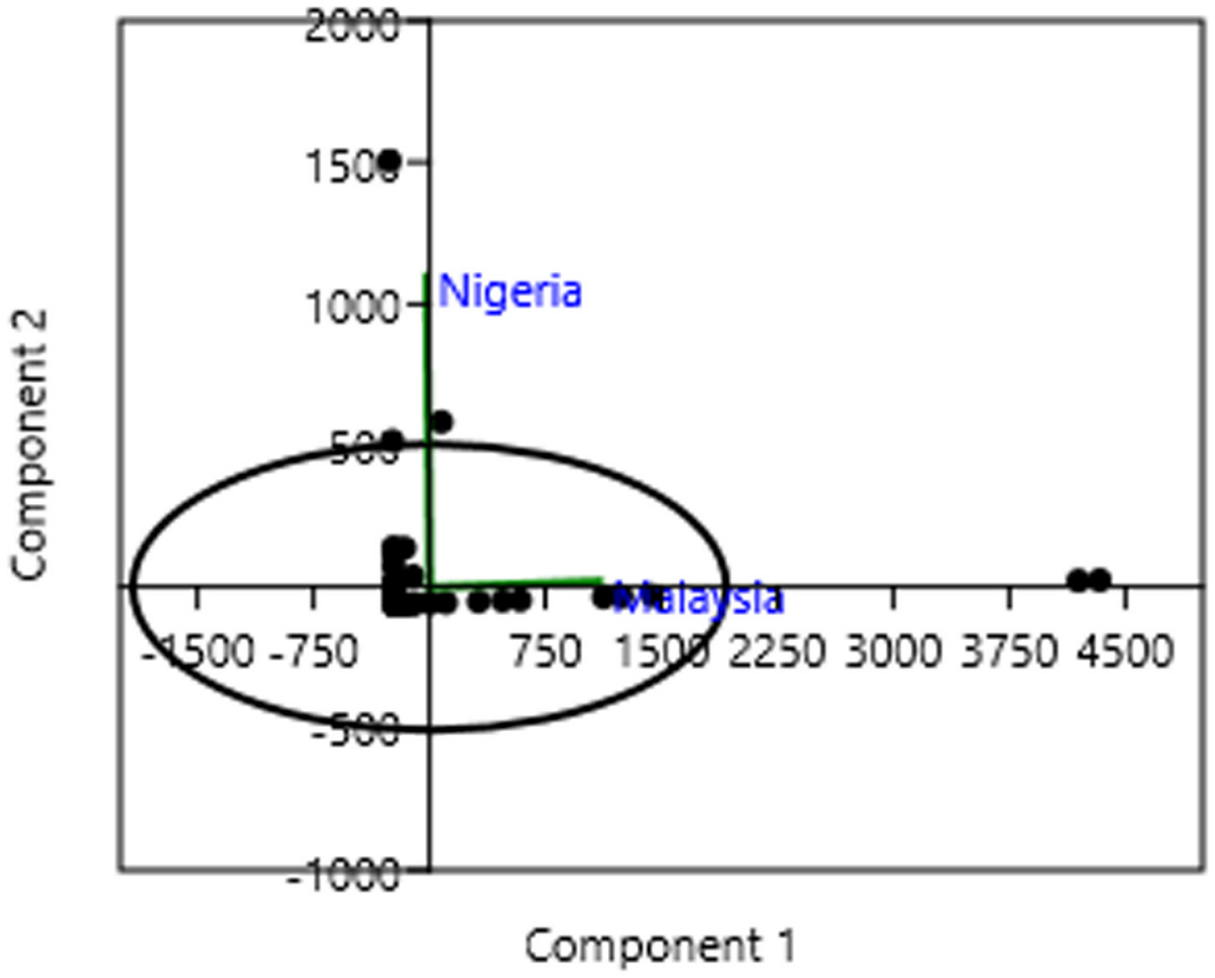
Principal component analysis showing the variations in the distribution of fern species in the two countries

It should be noted that some of these identified ferns exhibited more than one life form. For example, *Pteris vittata* existed as both terrestrial and lithophytic fern while *Nephrolepis biserrata* existed as both terrestrial and epiphytic fern in Malaysia and Nigeria. Jones et al. (2011) also reported that some ferns have the ability to exist in different life forms. The PCA showed that the principal components (PC) 1 and 2 contributed to 93.52% and 6.48% of the total variations, respectively (Figure 5). However, Malaysian forests contributed more significantly to the PC 1 and Nigerian forests contributed to PC 2. This invariably shows that Malaysian forests contributed largely to the overall variations in the fern species. *Pyrrosia lanceolata* and *Drynaria quercifolia* were observed to be the ferns with the highest relative frequencies of 25.36% and 24.59%, respectively, in the combined forests of Malaysia (Table 4). In Nigeria, *Pneumatopteris afra* with the relative frequency of 35.62% was observed to be the highest.

**TABLE 4:** The distribution of ferns observed in the study areas

S/N	Species	Family	Countries			
			Malaysia		Nigeria	
			Presence/absence	Relative frequency (%)	Presence/absence	Relative frequency (%)
1	*Acrosticum aureum*	Pteridaceae	✓	0.24	✗	0
2	*Adiantum capillus‐veneris*	Adiantaceae	✗	0	✓	0.27
3	*Adiantum latifolium*	Adiantaceae	✓	0.03	✗	0
4	*Angiopteris angustifolia*	Marattiaceae	✓	0.12	✗	0
5	*Angiopteris evecta*	Marattiaceae	✓	0.09	✗	0
6	*Asplenium batuense*	Aspleniaceae	✓	0.089	✗	0
7	*Asplenium nidus*	Aspleniaceae	✓	1.87	✗	0
8	*Asplenium scolopendria*	Aspleniaceae	✗	0	✓	0.97
9	*Asplenium scortechinii*	Aspleniaceae	✓	0.13	✗	0
10	*Asplenium trichomanes*	Aspleniaceae	✗	0	✓	0.97
11	*Bolbitis virens*	Lomariopsidaceae	✗	0.16	✗	0
12	*Bolbitis gemmifera*	Lomariopsidaceae	✗	0	✓	1.20
13	*Ceratopteris cornuta*	Parkeriaceae	✗	0	✓	0.52
14	*Cyathea contaminans*	Cyatheaceae	✓	0.09	✗	0
15	*Cyathea moluccana*	Cyatheaceae	✓	0.18	✗	0
16	*Cyclosorus ecallosa*	Thelypteridaceae	✓	0.13	✗	0
17	*Cyclopeltis crenata*	Aspidiaceae	✓	0.07	✗	0
18	*Davallia denticulata*	Davalliaceae	✓	3.89	✗	0
19	*Drymoglossum piloselloides*	Polypodiaceae	✓	4.53	✗	0
20	*Drynaria quercifolia*	Polypodiaceae	✓	24.59	✗	0
21	*Drynaria rigidula*	Polypodiaceae	✓	0.19	✗	0
22	*Drynaria sparsisora*	Polypodiaceae	✓	0.31	✗	0
23	*Elaphoglossum callifolium*	Lomariopsidaceae	✓	0.06	✗	0
24	*Gleichenia linearis*	Gleicheniaceae	✗	0	✓	1.79
25	*Gleichenia truncata*	Gleicheniaceae	✓	7.53	✗	0
26	*Goniophlebium verrucosum*	Polypodiaceae	✓	0.07	✗	0
27	*Hymenophyllum acanthoides*	Hymenophyllaceae	✓	0.05	✗	0
28	*Hymenophyllum productum*	Hymenophyllaceae	✓	0.06	✗	0
29	*Lepisorus longifolius*	Polypodiaceae	✓	0.03	✗	0
30	*Lindsaea lucida*	Lindsaeaceae	✓	1.29	✗	0
31	*Lindsaea napaea*	Lindsaeaceae	✓	8.18	✗	0
32	*Lygodium circinnatum*	Schizaeaceae	✓	0.05	✗	0
33	*Lygodium longifolium*	Schizaeaceae	✓	9.28	✗	0
34	*Lygodium japonicum*	Schizaeaceae	✗	0	✓	3.05
35	*Lygodium microphyllum*	Schizaeaceae	✗	0	✓	4.00
36	*Merinthosorus drynarioides*	Polypodiaceae	✓	0.25	✗	0
37	*Microgramma owariensis*	Polypodiaceae	✗	0	✓	1.00
38	*Nephrolepis acutifolia*	Nephrolepidaceae	✓	0.74	✗	0
39	*Nephrolepis biserrata*	Nephrolepidaceae	✓	1.76	✓	14.52
40	*Nephrolepis cordifolia*	Nephrolepidaceae	✗	0	✓	1.77
41	*Nephrolepis dicksonioides*	Nephrolepidaceae	✓	0.25	✗	0
42	*Nephrolepis duffii*	Nephrolepidaceae	✗	0	✓	0.97
43	*Nephrolepis exalta*	Nephrolepidaceae	✗	0	✓	1.52
44	*Nephrolepis furcans*	Nephrolepidaceae	✗	0	✓	0.14
45	*Nephrolepis tuberosa*	Nephrolepidaceae	✓	0.18	✗	0
46	*Nephrolepis undulata*	Nephrolepidaceae	✗	0	✓	13.12
47	*Orthiopteris kingii*	Dennstaedtiaceae	✓	0.07	✗	0
48	*Phymatodes scolopendria*	Polypodiaceae	✗	0	✓	0.57
49	*Phymatosorus nigrescens*	Polypodiaceae	✓	0.04	✗	0
50	*Pityrogramma calomelanos*	Hemionitidaceae	✓	0.69	✓	2.23
51	*Platycerium angolense*	Polypodiaceae	✗	0	✓	1.20
52	*Platycerium coronatum*	Polypodiaceae	✓	0.11	✗	0
53	*Platycerium stemaria*	Polypodiaceae	✗	0	✓	0.79
54	*Pneumatopteris afra*	Thelypteridaceae	✗	0	✓	35.62
55	*Pneumatopteris truncata*	Thelypteridaceae	✓	0.13	✗	0
56	*Pronephrium asperum*	Thelypteridaceae	✓	0.08	✗	0
57	*Pronephrium menisciicarpon*	Thelypteridaceae	✓	0.34	✗	0
58	*Pronephrium salicifolium*	Thelypteridaceae	✓	0.19	✗	0
59	*Pronephrium triphyllum*	Thelypteridaceae	✓	0.29	✗	0
60	*Pteridium aquilinum*	Hypolepidaceae	✗	0	✓	4.59
61	*Pteris acanthoneura*	Pteridaceae	✗	0	✓	1.20
62	*Pteris atrovirens*	Pteridaceae	✗	0	✓	0.77
63	*Pteris burtoni*	Pteridaceae	✗	0	✓	1.50
64	*Pteris mildbraedii*	Pteridaceae	✗	0	✓	0.50
65	*Pteris semipinnata*	Pteridaceae	✓	0.08	✗	0
66	*Pteris venulosa*	Pteridaceae	✓	0.13	✗	0
67	*Pteris vittata*	Pteridaceae	✓	0.39	✓	4.50
68	*Pteridium caudatum*	Pteridaceae	✓	0.29	✗	0
69	*Pyrrosia lanceolata*	Polypodiaceae	✓	25.36	✗	0
70	*Salvinia molesta*	Salviniaceae	✓	0.25	✗	0
71	*Scleroglossum minus*	Grammitidaceae	✓	3.04	✗	0
72	*Tectaria barberi*	Aspidiaceae	✓	0.29	✗	0
73	*Tectaria crenata*	Aspidiaceae	✓	0.36	✗	0
74	*Tectaria singaporeana*	Aspidiaceae	✓	0.68	✗	0
75	*Thelypteris paleata*	Thelypteridaceae	✓	0.12	✗	0
76	*Thelypteris viscosa*	Thelypteridaceae	✓	0.07	✗	0
77	*Trichomanes humile*	Hymenophyllaceae	✓	0.43	✗	0
78	*Vittaria guinensis*	Vittariaceae	✗	0	✓	0.66

Note

✓ means present, ✗ means absent.

The common fern species shared by the two countries include *Nephrolepis biserrata*, *Pityrogramma calomelanos*, and *Pteris vittata*. This makes the Sorenson similarity index of the fern species between the two countries to be 7.41%. This is a very low index of similarity which indicates that the two countries, although within the tropics, do not share many fern species in common. This may be due to differences in their evolutionary or historical processes, plus climatic factors of the two regions (Zelazowski et al., 2011). However, the similarity in the ferns composition between the two countries increased as the taxonomic level increased (species: 7.41%, genus: 12.77%, family: 70.96%). The family Polypodiaceae represented by seven genera and nine species is the dominant family of ferns in Malaysia, whereas Nephrolepidaceae represented by one genus and six species is the dominant family in Nigeria (Table 5). The dominance of the family Polypodiaceae in Peninsular Malaysia has also been reported by previous studies (Rahmad & Akomolafe, 2018, 2019).

**TABLE 5:** Checklist of families with respective numbers of general and species

S/N	Family	Malaysia		Nigeria	
		Number of genera	Number of species	Number of genera	Number of species
1	Adiantaceae	1	1	1	1
2	Aspleniaceae	1	3	1	2
3	Aspidiaceae	2	4	0	0
4	Cyatheaceae	1	2	0	0
5	Davalliaceae	1	1	0	0
6	Dennstaedtiaceae	1	1	0	0
7	Gleicheniaceae	1	1	1	1
8	Grammitidaceae	1	1	0	0
9	Hemionitidaceae	1	1	1	1
10	Hymenophyllaceae	2	3	0	0
11	Hypolepidaceae	0	0	1	1
12	Lindsaeaceae	1	2	0	0
13	Lomariopsidaceae	2	3	1	1
14	Marattiaceae	1	2	0	0
15	Nephrolepidaceae	1	4	1	6
16	Parkeriaceae	0	0	1	1
17	Polypodiaceae	7	9	3	4
18	Pteridaceae	2	5	1	5
19	Salviniaceae	1	1	0	0
20	Schizaeaceae	1	2	1	2
21	Thelypteridaceae	4	8	1	1
22	Vittariaceae	0	0	1	1

### 3.4 The climatic drivers of the species richness and phylogenetic diversity of ferns

The phylogenetic diversity of ferns in Malaysia (PD = 2.574) was found to be higher than that of Nigeria (PD = 0.182). The higher diversity and richness of ferns in Malaysia as compared with Nigerian (in terms of PD and SR) can be attributed to the ancestral niche hypothesis which predicted that higher diversity of ferns are in the ancestral niche due to accumulation of taxa and inablility of the ferns to move to other niches (Wiens et al., 2010). Other historical factors such as the reduced extinction rates, large geographical extent of the rainforest, and age of the tropic could also explain the higher diversity of ferns observed in the tropic regions (Qian & Ricklefs, 2004). All these are likely showing that Malaysia is the ancestral home of ferns as compared with Nigeria.

In Nigeria, the ferns species richness was most strongly correlated with minimum temperature of coldest month, mean annual temperature, and precipitation of driest month (*r* = .95, .99, and .99, respectively, Table 6). However, the relationships between the phylogenetic diversity and the bioclimatic variables were weak except for precipitation seasonality and annual precipitation (*r* = .98 and 1.00, respectively). Also in Malaysia, the SR is driven strongly by the precipitation of the driest month and annual precipitation (*r* = .96 and .98 respectively). As for the PD of ferns in Malaysia, it is driven strongly by most of the bioclimatic variables. It is worthy to note that all the relationships between the bioclimatic variables, PD and SR were positive. The pattern of the ferns assemblages in Nigeria and Malaysia revealed that both temperature and precipitation variables play vital roles.

**TABLE 6:** The correlation coefficients of the relationships between the bioclimatic variables, PD and SR

Bioclimatic variable	Nigeria		Malaysia	
	PD	SR	PD	SR
Mean annual temperature (Bio1)	0.65	0.99	0.94	0.85
Temperature seasonality (Bio4)	0.50	0.94	0.97	0.77
Minimum temperature of coldest month (Bio6)	0.50	0.95	0.76	0.87
Annual precipitation (Bio12)	1.00	0.75	0.99	0.98
Precipitation of driest month (Bio14)	0.82	0.99	0.89	0.96
Precipitation seasonality (Bio15)	0.98	0.87	0.96	0.65

The precipitation variables drive the phylogenetic structure of ferns in Nigeria, whereas both precipitation and temperature variables drive the phylogenetic structure of ferns in Malaysia. Water has been reported as the leading determinant of the richness of ferns across the world (Nagalingum et al., 2014). This is buttressed by the pattern observed in this study whereby both PD and SR were strongly influenced by the precipitation variables in Malaysia and Nigeria. Researchers have identified precipitation, elevation, and temperature variables as the most important determinants of ferns richness and other types of plants at global scale (Kreft & Jetz, 2007; Kreft et al., 2010). This explains the higher richness of ferns in wet tropical areas, particularly with reference to increasing elevation (Kessler, 2010; Kreft et al., 2010).

## 4 CONCLUSION

The results indicated that the Malaysian forests studied provide favorable microclimatic conditions which supported significantly higher fern diversity and richness than those Nigerian forests. There are distinct differences in the fern community composition within each country and between the two countries which led to a very low similarity index at the species and genus levels. These observed differences in the species composition and phylogenetic diversity are explained by different bioclimatic variables particularly the temperature and precipitation variables. This may as well be due to other historical and evolutionary processes between the two biogeographic areas. We thereby recommend that future researches should include forests of other countries to have a robust comparison of variations in ferns community assemblages between Asia and Africa. We also recommend a greater number of sites to be sampled over an elevational gradient to capture sufficient variation in environment across the two continents.

## AUTHOR CONTRIBUTIONS

**Gbenga F. Akomolafe:** Conceptualization (lead); Data curation (equal); Funding acquisition (supporting); Methodology (equal); Writing – review & editing (equal). **Rusly Rosazlina:** Conceptualization (supporting); Funding acquisition (lead); Investigation (equal); Resources (equal); Writing – review & editing (equal). **Zakaria Rahmad:** Project administration (equal); Supervision (lead); Writing – original draft (equal). **Fatai A. Oloyede:** Supervision (equal); Writing – original draft (equal); Writing – review & editing (equal).

## CONFLICT OF INTEREST

The authors hereby declare that there is no conflict of interest in this work.

## Supporting information

Appendix S1‐S2

## Data Availability

The diversity data and final protein sequence assembly have been deposited in Dryad (https://doi.org/10.5061/dryad.tx95x6b10).
