# Supplementary material for: Patterns of ferns community assemblages in some Malaysian and Nigerian tropical forests
Source: Ecol Evol. 2022 Jun 3;12(6):e8961. doi: 10.1002/ece3.8961 (PMC9165205; doi:10.1002/ece3.8961)
Supplement: Supplementary file 1 — Appendix S1‐S2 [file ECE3-12-e8961-s001.docx]

**APPENDICES**


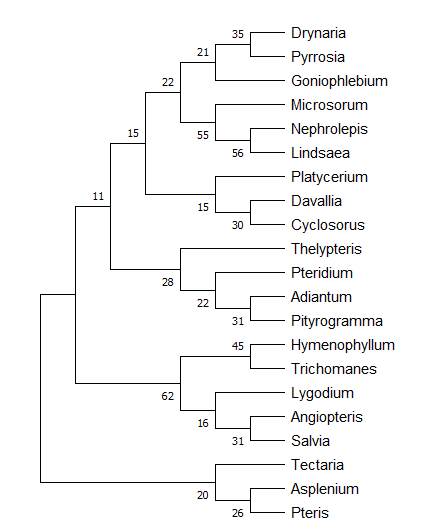


Appendix 1: The phylogenetic tree for Malaysian ferns


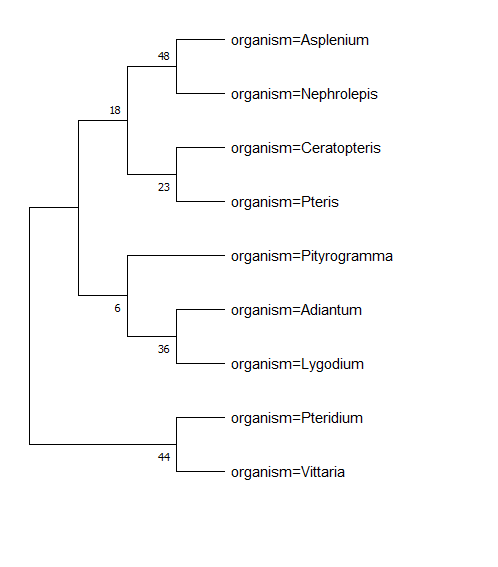


Appendix 2: The phylogenetic tree for Nigerian ferns
